# Organizing Pneumonia Secondary to Pulmonary Actinomycosis: A Case Report and Literature Review

**DOI:** 10.7759/cureus.21133

**Published:** 2022-01-11

**Authors:** Sohaib Khatib, Ahmad Al-Shyoukh,, Khalid Abdalla, Fouad S Jaber, Gary Salzman

**Affiliations:** 1 Internal Medicine, University of Missouri Kansas City School of Medicine, Kansas City, USA; 2 Pulmonary and Critical Care Medicine, Tulane University School of Medicine, New Orleans, USA; 3 Pulmonary and Critical Care Medicine, University of Missouri Kansas City School of Medicine, Kansas City, USA

**Keywords:** organizing pneumonia, hypoxia, actinomyces, respiratory failure, pulmonary actinomycosis

## Abstract

Pulmonary actinomycosis is a common clinical infection caused by *Actinomyces* species. Although its treatment is very effective with Intravenous (IV) antibiotics, its diagnosis is challenging and easily missed. Organizing Pneumonia (OP) can be cryptogenic or secondary to different clinical diseases. Herein, we discuss a case of acute hypoxemic respiratory failure that was found to be due to OP, secondary to pulmonary actinomycosis, with a brief review of the literature. A 64-year-old male presented with acute hypoxemic respiratory failure two days after undergoing elective right total hip arthroplasty. Chest imaging with CT scan showed symmetric bilateral ground-glass opacities most pronounced within the upper lung lobes. The patient was treated initially with IV diuresis, steroids, and broad-spectrum antibiotics. However, his clinical status continued to worsen and his chest imaging showed worsening lung opacities. Video-assisted thoracoscopic lung biopsy (VATS) was done, and pathology results showed features of organizing pneumonia. Tissue culture confirmed *Actinomyces* species. The patient had clinical improvement after treatment with IV methylprednisolone and IV penicillin G.

Pulmonary actinomycosis is very rarely associated with OP but this bacterial infection should always be in the differential diagnosis when OP is confirmed as the treatment is effective with IV antibiotics.

## Introduction

*Actinomyces* spp are gram-positive, facultative, anaerobic, rod-shaped bacteria that are normal flora in the oropharynx and gastrointestinal tract, and can become pathogenic once the integrity of the mucosal barrier is disrupted [1]. The three most common human actinomyces clinical infections are cervicofacial, abdominopelvic, and pulmonary [2]. Pulmonary actinomycosis is usually diagnosed late or misdiagnosed with other chronic suppurative lung infections or malignancy. One reason for that is that *Actinomyces* is sensitive to common antibiotics that are usually started for other reasons [2].

Organizing pneumonia (OP) is a particular type of lung reaction to an inflammatory stimulus that results in the accumulation of fibrin and myxoid matrix in alveolar spaces. It can be secondary to several conditions such as connective tissue diseases, immunodeficiency syndromes, lung and bone marrow transplant, toxic fumes, viral or bacterial infections [3]. In this report, we discuss a case of pulmonary actinomycosis along with OP resulting in hypoxemic respiratory failure.

## Case presentation

**Figure 1:**
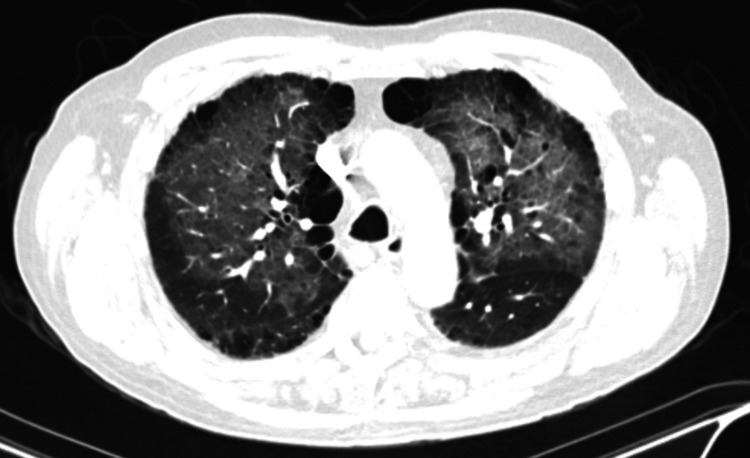
Chest CT scan showing symmetric bilateral ground-glass opacities with associated interstitial thickening within a perihilar distribution most pronounced within the upper lobes in addition to mild para-septal emphysematous changes, and bilateral subpleural reticular opacities suspicious for underlying fibrotic changes

A 64-year-old male patient with a past medical history of mild chronic obstructive pulmonary disease (COPD), hypertension, alcohol use, tobacco use, and degenerative joint disease was admitted initially to an outside hospital where he underwent right total hip arthroplasty under general anesthesia on day one. He did not have any complications during surgery. However, on day two, he started to develop respiratory symptoms with shortness of breath and productive cough and was found to have hypoxemia with increased oxygen requirement. His oxygen (O_2_) saturation dropped down to 75% on room air, and supplemental oxygen with 2 liters/minute by nasal cannula was provided. The patient’s other vital signs were within normal limits. Chest x-ray (CXR) done at that time showed mild cardiomegaly with diffuse bilateral reticular and interstitial opacities. Given concerns for pulmonary embolism (PE), he underwent CT angiography of his chest, which was negative for any evidence of pulmonary embolism. However, it showed symmetric bilateral ground-glass opacities with associated interstitial thickening within a perihilar distribution most pronounced within the upper lobes. It also showed mild para-septal emphysematous changes, mediastinal and bilateral hilar adenopathy, and bilateral subpleural reticular opacities suspicious for underlying fibrotic changes (Figure 1).

Initially, he was started on intravenous (IV) furosemide with a dose of 40 mg twice daily and albuterol-ipratropium nebulizer treatments for likely pulmonary edema or COPD exacerbation. A trans-thoracic echocardiogram was done and showed normal ejection fraction (60-65%) with normal diastolic function and no wall motion abnormalities. His oxygen requirements increased up to 10 liters/minute and he was then placed on noninvasive positive pressure ventilation (NIPPV) for worsening hypoxic respiratory failure and was transferred to the ICU. Diuresis, steroids, and inhaled bronchodilators were continued. Blood, urine, and sputum cultures were sent and came back negative. Urine *Legionella* and *Streptococcus pneumoniae* antigens were negative as well. Procalcitonin was 0.14 ng/ml (normal level is <0.15 ng/ml). He was transferred to the medicine floor after five days with improvement in hypoxic respiratory failure.

**Figure 2:**
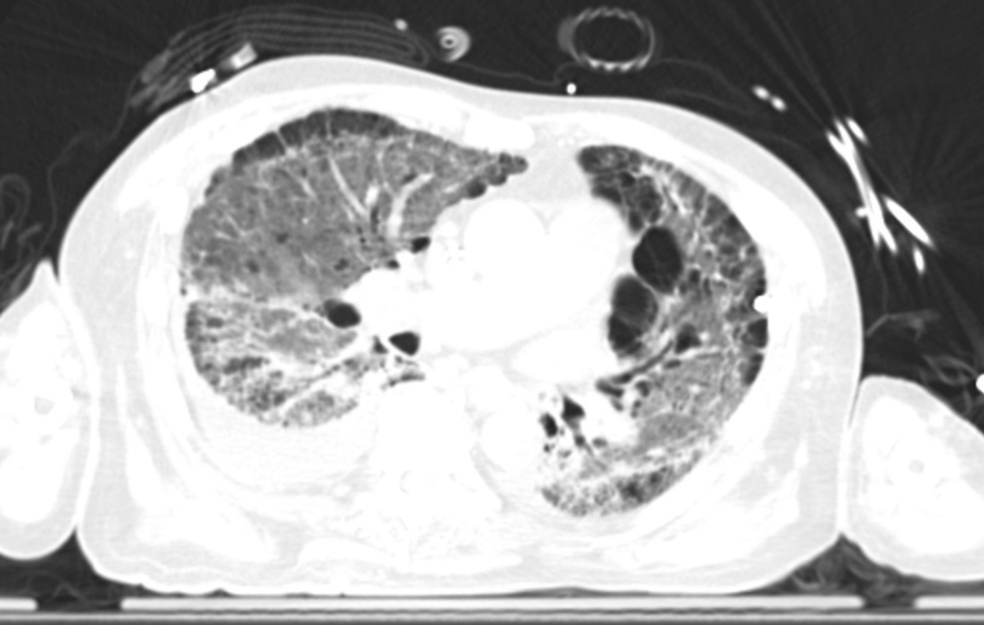
Repeat chest CT scan showing the progression of ground-glass opacities and reticulation in bilateral upper lobes and lower lobes, with worsening diffuse bilateral subpleural reticular opacities with the suggestion of honeycombing

Two days after transfer out of ICU, he developed a fever of 102.3 Fahrenheit, and repeat CXR showed mild worsening of left-sided heterogeneous opacities. Therefore, IV antibiotics with piperacillin-tazobactam and vancomycin were started for treatment of presumed hospital-acquired pneumonia (HAP). Vancomycin was stopped after three days. A repeat CT chest showed progression of ground-glass opacities and reticulation in bilateral upper lobes and lower lobes, with worsening diffuse bilateral subpleural reticular opacities with the suggestion of honeycombing (Figure 2).

Further work-up with a rheumatological panel with antineutrophil antibody, anticentromere antibody, rheumatoid factor, anti-scl-70 antibody, antineutrophil cytoplasmic antibody, and anti-Jo-1, all were negative. Antibiotics were transitioned to oral Augmentin and diuresis was stopped.

Due to continued worsening in his clinical status in addition to concerning chest imaging findings, the plan was to proceed with video-assisted thoracoscopic lung biopsy (VATS). VATS with right lung biopsy was performed on day 19 of hospitalization. The patient had a chest tube placement for a small right-sided pneumothorax as a complication of lung biopsy. He failed the spontaneous breathing trial with worsening respiratory acidosis and increased oxygen requirements on mechanical ventilation. Augmentin was discontinued after completing a seven-day course. However, his respiratory status continued to worsen. The patient’s blood pressure dropped down after this requiring intermittent vasopressor support with norepinephrine.

**Figure 3:**
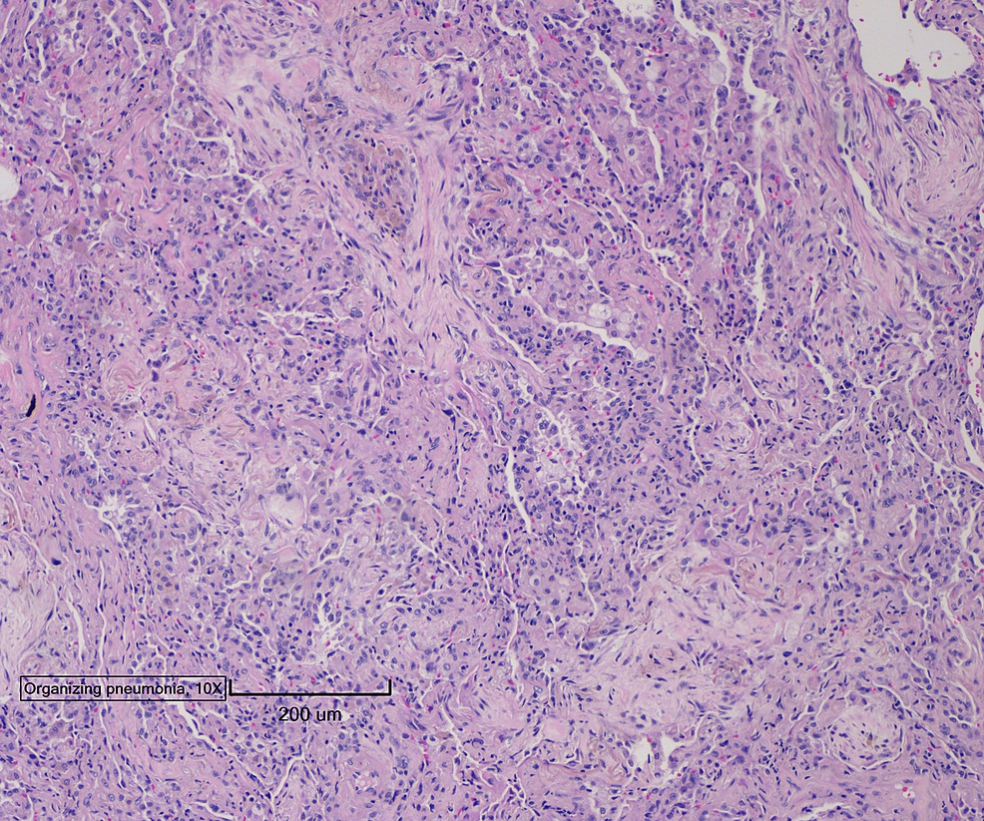
Lung biopsy pathology image showing features of organizing pneumonia, bronchial squamous metaplasia with interstitial fibrosis, and emphysema

**Figure 4:**
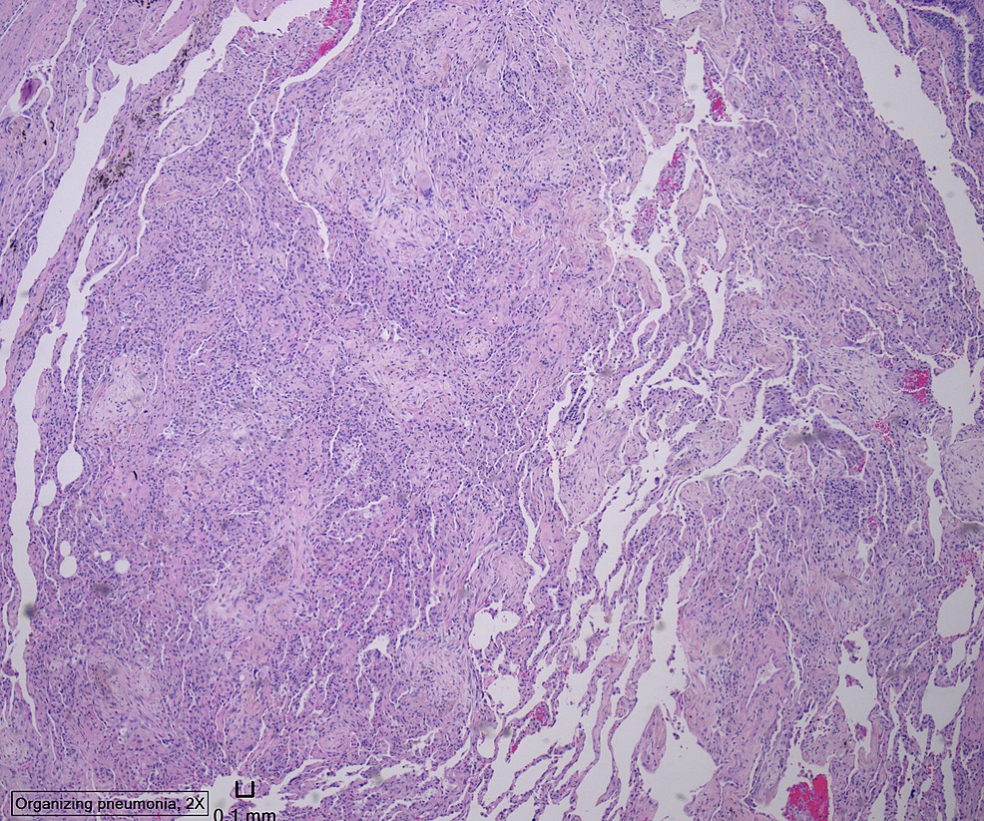
Lung biopsy pathology image showing features of organizing pneumonia, bronchial squamous metaplasia with interstitial fibrosis, and emphysema

**Figure 5:**
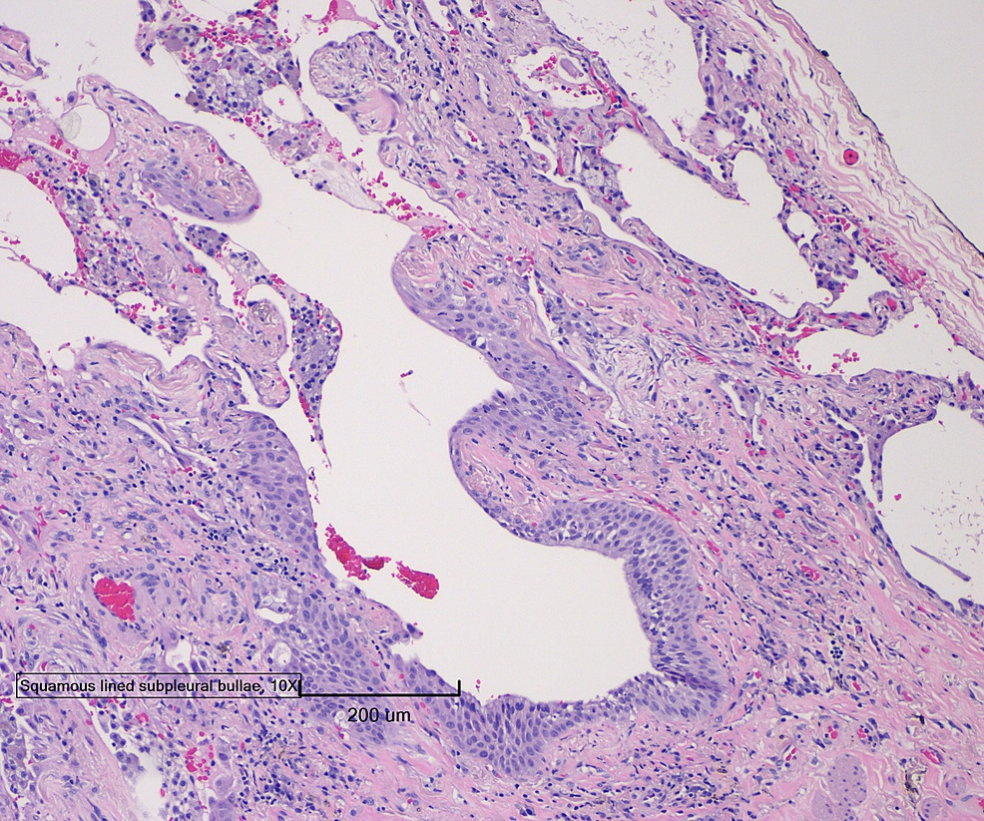
Lung biopsy pathology image showing features of organizing pneumonia, bronchial squamous metaplasia with interstitial fibrosis, and emphysema

Four days after lung biopsy was done, pathology results showed features of OP, bronchial squamous metaplasia with interstitial fibrosis, and emphysema (Figure 3-5).

Subsequently, the patient was started on IV methylprednisolone 1 gram daily for a five-day course and he was successfully weaned off ventilator settings. On day 25 of hospitalization, lung biopsy culture from two samples started growing gram-positive rods; therefore, the patient was started on ampicillin-sulbactam. Two days later, the final culture report confirmed *Actinomyces* spp, so he was switched to IV penicillin G at a dose of 4 million units every four hours, for a total of six weeks, to be followed by 12 months of penicillin V. He was also continued on IV methylprednisolone 60 mg daily after finishing the five-day course of IV methylprednisolone 1 gram daily. The patient was then discharged to a long-term acute care facility to continue long-term IV antibiotics.

## Discussion

We presented a case of OP secondary to *Actinomyces* pulmonary infection. Around 15% of human actinomycosis infections manifest as pulmonary infection [4]. Major risk factors for pulmonary actinomycosis include aspiration of gastric or oropharyngeal contents, poor dental hygiene, diabetes, alcoholism, and structural lung diseases [5]. Pulmonary actinomycosis can present as acute or subacute infection followed by chronic stage with several nonspecific respiratory symptoms including productive cough, fever, shortness of breath, chest pain, hemoptysis, night sweats, and weight loss [6]. Diagnosis of pulmonary actinomycosis is challenging due to sharing several clinical and radiological findings with other common lung diseases especially malignancy, suppurative lung infections, and tuberculosis. Therefore, it usually takes around six months on average to reach a definitive diagnosis [7]. Pulmonary actinomycosis can manifest radiologically with several non-specific features including consolidation, nodular or mass-like lesions, pleural thickening, and lymphadenopathy [8]. Reaching a diagnosis requires a combination of clinical, radiological features, microbiological evidence with culture-positive bronchoalveolar secretions, sputum, blood, or pleural effusion, and also pathological evidence on biopsy with characteristic sulfur granules. Despite the challenging diagnosis process, actinomycosis is sensitive to many commonly used antibiotics in clinical practice. The treatment of choice for pulmonary actinomycosis is high-dose intravenous penicillin for a long duration of 6-12 months [2].

OP, previously named bronchiolitis obliterans organizing pneumonia (BOOP), is a rare disease with an incidence of 1.96 per 100,000 [9]. Clinically, the disease usually manifests with several weeks of dyspnea, weight loss, fatigue, and dry cough with worsening dyspnea being the major issue for most patients. However, it can also present as acute respiratory failure [10]. The most common pattern on radiologic imaging is peripheral bilateral diffuse alveolar opacities predominantly in lower lung lobes. OP is characterized by a restrictive pattern with decreased diffusion capacity for carbon monoxide on spirometry [11]. To confirm the diagnosis, histopathologic examination of a tissue sample obtained by transbronchial lung biopsy, open lung biopsy, or video-assisted thoracoscopy is usually required [11]. The standard therapy for OP and subsequent flares is corticosteroids; in addition to that, treatment of underlying disease associated with secondary OP is also required for complete resolution.

Our patient developed hypoxemic respiratory failure secondary to pulmonary actinomycosis. The etiology of this is multifactorial, but given his presentation, it is likely that this was secondary to *Actinomyces* infection, likely from aspiration. Aspiration during intubation and mechanical ventilation remains the main risk factor, which is the major route for *Actinomyces* normal flora to cause invasive lung infection [11]. It is also likely that this patient has had an aspiration event prior to the presentation given his history of significant alcohol use. His CXR and chest CT scan showed diffuse bilateral ground-glass opacities more pronounced in the upper lobes, which is atypical for organizing pneumonia, which usually predominates in lower lung lobes [12]. During his prolonged hospitalization, our patient was treated with several forms of corticosteroids with oral prednisone and IV methylprednisolone; this was given initially for presumptive COPD exacerbation. However, he did not show improvement on lower doses of steroids alone although corticosteroids are the treatment of choice for organizing pneumonia. He showed improvement when he was started on both *Actinomyces*-directed antibiotics and high dose corticosteroids after diagnosis confirmed for both pulmonary actinomycosis and OP, this emphasizes the importance of treating the underlying secondary cause of OP for the lung disease to be adequately treated [12].

Secondary OP can be caused by several bacterial organisms, these commonly include *Streptococcus pneumoniae*, *Legionella pneumophila, Mycoplasma pneumonia, Nocardia asteroides*, and *Staphylococcus aureus*. It is very unusual for *Actinomyces* to be associated with OP. In reviewing the literature, there are only two reported cases of OP associated with pulmonary actinomycosis [13,14]. Our patient showed acute chronic worsening of his respiratory status; however, the patients in the two other reported cases manifested chronic respiratory and constitutional symptoms. Our patient and the patient in the report by Alfaro et al. [13] had the aspiration of oropharyngeal contents as the major risk factor. Findings on chest imaging were variable in the three cases. While our patient showed bilateral ground-glass opacities, the chest imaging in the report by Alfaro et al. [13] was very concerning with mass findings. In a report by Fujita et al., the patient showed bilateral consolidations and air bronchograms on his chest imaging [14]. It is interesting that *Actinomyces* was detected in three different methods in these reported cases; in our case, it was detected by an anaerobic culture, which is the standard method of diagnosis. In the case reported by Alfaro et al. [13], it was diagnosed with the histopathological observation of *Actinomyces* colonies. The method used for *Actinomyces* *graevenitzii* detection in the case reported by Fujita et al. [14] was polymerase chain reaction (PCR) and 16S rRNA gene sequencing after failed detection on culture and histological observation. Our patient and the patient reported by Fujita et al. [14] were both treated with antibiotics and corticosteroids; however, the patient described by Alfaro et al. [13] was cured with surgical lobectomy alone (Table 1). 

**Table 1:** Literature review: comparison between three cases of organizing pneumonia (OP) associated with pulmonary actinomycosis PCR: polymerase chain reaction

Case/ face of comparison	Presentation	Risk factor	Imaging findings	Lung biopsy method and findings	Actinomyces diagnosis	Treatment
Current Case	acute on chronic	aspiration/recent surgery	bilateral ground-glass opacities	video-assisted thoracoscopic lung biopsy-pattern of organizing pneumonia with interstitial fibrosis	lung biopsy culture	IV penicillin G and IV corticosteroids
Alfaro et al., 2011 [13]	chronic	dental prosthesis and several dental caries	mass in right lung lower lobe	transthoracic biopsy- a pattern of organizing pneumonia with giant multinucleated cell granulomas	histological observation	surgical resection
Fujita et al., 2012 [14]	chronic	undetermined	bilateral consolidations with air bronchograms	video-assisted thoracoscopic lung biopsy- a pattern of organizing pneumonia with microabscesses	PCR and gene sequencing of bronchial specimen	oral corticosteroid and IV ampicillin

## Conclusions

While several lung bacterial infections can present in association with OP, one very unusual cause is pulmonary *Actinomyces* infection. This should be included in the differential diagnosis for secondary causes of OP. Having a high degree of suspicion and reaching the correct diagnosis will result in appropriate, timely management.

## References

[1] Wong VK, Turmezei TD, Weston VC (2011). Actinomycosis. BMJ.

[2] Mabeza GF, Macfarlane J (2003). Pulmonary actinomycosis. Eur Respir J.

[3] Alasaly K, Muller N, Ostrow DN, Champion P, FitzGerald JM (1995). Cryptogenic organizing pneumonia. A report of 25 cases and a review of the literature. Medicine (Baltimore).

[4] Skehan N, Naeem M, Reddy RV (2015). Endobronchial actinomycosis: successful treatment with oral antibiotics. BMJ Case Rep.

[5] Gupta P, Dogra V, Goel N, Chowdhary A, Prasad R, Gaur SN (2015). An unusual cause of a pulmonary mass: actinomycosis. Indian J Chest Dis Allied Sci.

[6] Zhang M, Zhang XY, Chen YB (2017). Primary pulmonary actinomycosis: a retrospective analysis of 145 cases in mainland China. Int J Tuberc Lung Dis.

[7] Weese WC, Smith IM (1975). A study of 57 cases of actinomycosis over a 36-year period. A diagnostic 'failure' with good prognosis after treatment. Arch Intern Med.

[8] Kwong JS, Müller NL, Godwin JD, Aberle D, Grymaloski MR (1992). Thoracic actinomycosis: CT findings in eight patients. Radiology.

[9] Gudmundsson G, Sveinsson O, Isaksson HJ, Jonsson S, Frodadottir H, Aspelund T (2006). Epidemiology of organising pneumonia in Iceland. Thorax.

[10] Nizami IY, Kissner DG, Visscher DW, Dubaybo BA (1995). Idiopathic bronchiolitis obliterans with organizing pneumonia. An acute and life-threatening syndrome. Chest.

[11] Apothéloz C, Regamey C (1996). Disseminated infection due to Actinomyces meyeri: case report and review. Clin Infect Dis.

[12] Drakopanagiotakis F, Polychronopoulos V, Judson MA (2008). Organizing pneumonia. Am J Med Sci.

[13] Alfaro TM, Bernardo J, Garcia H, Alves F, Carvalho L, Caseiro Alves F, Robalo Cordeiro C (2011). Organizing pneumonia due to actinomycosis: an undescribed association. Respiration.

[14] Fujita Y, Iikura M, Horio Y, Ohkusu K, Kobayashi N (2012). Pulmonary Actinomyces graevenitzii infection presenting as organizing pneumonia diagnosed by PCR analysis. J Med Microbiol.

